# Rapidity gap survival in enhanced Pomeron scheme

**DOI:** 10.1140/epjc/s10052-018-5564-z

**Published:** 2018-01-24

**Authors:** Sergey Ostapchenko, Marcus Bleicher

**Affiliations:** 1 Frankfurt Institute for Advanced Studies, 60438 Frankfurt am Main, Germany; 2 D.V. Skobeltsyn Institute of Nuclear Physics, Moscow State University, Moscow, 119992 Russia; 3 Institute for Theoretical Physics, Goethe-Universitat, 60438 Frankfurt am Main, Germany

## Abstract

We apply the phenomenological Reggeon field theory framework to investigate rapidity gap survival (RGS) probability for diffractive dijet production in proton–proton collisions. In particular, we study in some detail rapidity gap suppression due to elastic rescatterings of intermediate partons in the underlying parton cascades, described by enhanced (Pomeron–Pomeron interaction) diagrams. We demonstrate that such contributions play a subdominant role, compared to the usual, so-called “eikonal”, rapidity gap suppression due to elastic rescatterings of constituent partons of the colliding protons. On the other hand, the overall RGS factor proves to be sensitive to color fluctuations in the proton. Hence, experimental data on diffractive dijet production can be used to constrain the respective model approaches.

## Introduction

An important direction in experimental studies of high energy hadronic collisions is related to diffractive hadron production, in particular, to production of high transverse momentum $p_\mathrm{t}$ particles in events characterized by large rapidity gaps (RGs) not covered by secondary hadrons. The scientific interest to such so-called hard diffraction phenomena is multifold and related, in particular, to searches for signatures of new physics in a relatively clean experimental environment (see Ref. [1] for a recent review). On the other hand, the corresponding observables involve both perturbative and nonperturbative physics and may thus shed some light on the interplay of the two and provide an additional insight into the nonperturbative proton structure.

In contrast to hard diffractive processes in deep inelastic scattering, final states with large rapidity gaps constitute a much smaller fraction of events containing high $p_\mathrm{t}$ particles in proton-proton collisions. This is because hard processes typically take place for small values of the impact parameter *b* between the colliding protons, where one has a significant overlap of the projectile and target parton clouds, but then, also the probability for additional inelastic rescatterings between protons’ constituents is high. Therefore, there is little chance that a rapidity gap produced in a hard diffraction process at small *b* is not covered by secondaries created by the accompanying multiple scattering [2]. It has been realized long ago that the corresponding penalty factor, nicknamed “rapidity gap survival (RGS) probability”, results from an interplay between the transverse profile for a hard diffraction process of interest and the much broader inelastic profile for *pp* collisions [3].

Since then, the problem has been widely addressed in literature and numerous estimations of the RGS probability for various hard diffraction reactions have been obtained [4–22]. Most of those studies have been devoted to the dominant, so-called “eikonal”, mechanism of the RG suppression, related to elastic rescatterings between constituent partons of the colliding protons, addressing, in particular, the energy dependence of the RGS probability [5, 6] and the role of the inelastic diffraction treatment in respective models^1^ [6, 7, 11, 14]. Much less understood are the noneikonal absorptive effects corresponding to elastic rescatterings of intermediate partons, for which the obtained numerical results differ considerably [8, 9, 14].

In this work, we are going to investigate the RGS probabilities for diffractive dijet production in the framework of the Gribov’s Reggeon Field Theory (RFT) [23], addressing, in particular, in some detail the role of the noneikonal absorption. Our choice was partly motivated by previous study of soft diffraction by one of us, where such noneikonal effects proved to be extremely important, giving rise to huge (up to an order of magnitude) corrections to diffractive cross sections [24] (see, e.g., Fig. 15 in that reference). Since the role of semihard processes, for relatively small parton transverse momentum, in multiple scattering is not too different from the one of purely soft interactions, at least in our model, we expected that the noneikonal absorption is quite important for diffractive jet production as well.

More specifically, we employ the enhanced Pomeron framework [25–29], as implemented in the QGSJET-II model [30, 31]. The approach treats consistently both the usual multiple scattering processes, describing individual parton cascades as Pomeron exchanges, and rescatterings of intermediate partons in those cascades off the projectile and target protons and off each other, which is treated as Pomeron–Pomeron interactions. Importantly, the latter contributions are resummed to all orders [28, 29].

Hard processes are incorporated in the scheme following the so-called “semihard Pomeron” approach [32, 33]: splitting general parton cascades into soft and hard parts. The latter are characterized by high enough parton virtualities $|q^{2}|>Q_{0}^{2}$, $Q_{0}$ being some cutoff for pQCD being applicable, and are treated by means of the Dokshitzer–Gribov–Lipatov–Altarelli–Parisi (DGLAP) evolution equations. In turn, the nonperturbative soft parts involve low-$q^2$ ($|q^{2}|<Q_{0}^{2}$) partons and are described by phenomenological soft Pomeron asymptotics.

To treat low mass diffraction and the related absorptive effects, a Good–Walker-type [34] framework is employed, considering the interacting protons to be represented by a superposition of a number of eigenstates which diagonalize the scattering matrix, characterized by different couplings to Pomerons [35]. The respective partonic interpretation is based on the color fluctuations picture [36], i.e. the representation of the proton wave function by a superposition of parton Fock states of different sizes. Fock states of larger transverse size are characterized by lower (more dilute) spatial parton densities, while more compact ones are more densely packed with partons.^2^ As will be demonstrated in the following, such color fluctuations have an important impact on the strength of the rapidity gap suppression.

The outline of the paper is as follows. In Sect. 2, we derive expressions for cross sections of single and central diffractive dijet production, introducing step by step the various absorptive corrections. In Sect. 3, we present our numerical results and discuss them in some detail. Finally, we conclude in Sect. 4.

## Cross sections for diffractive dijet production

**Fig. 1:**
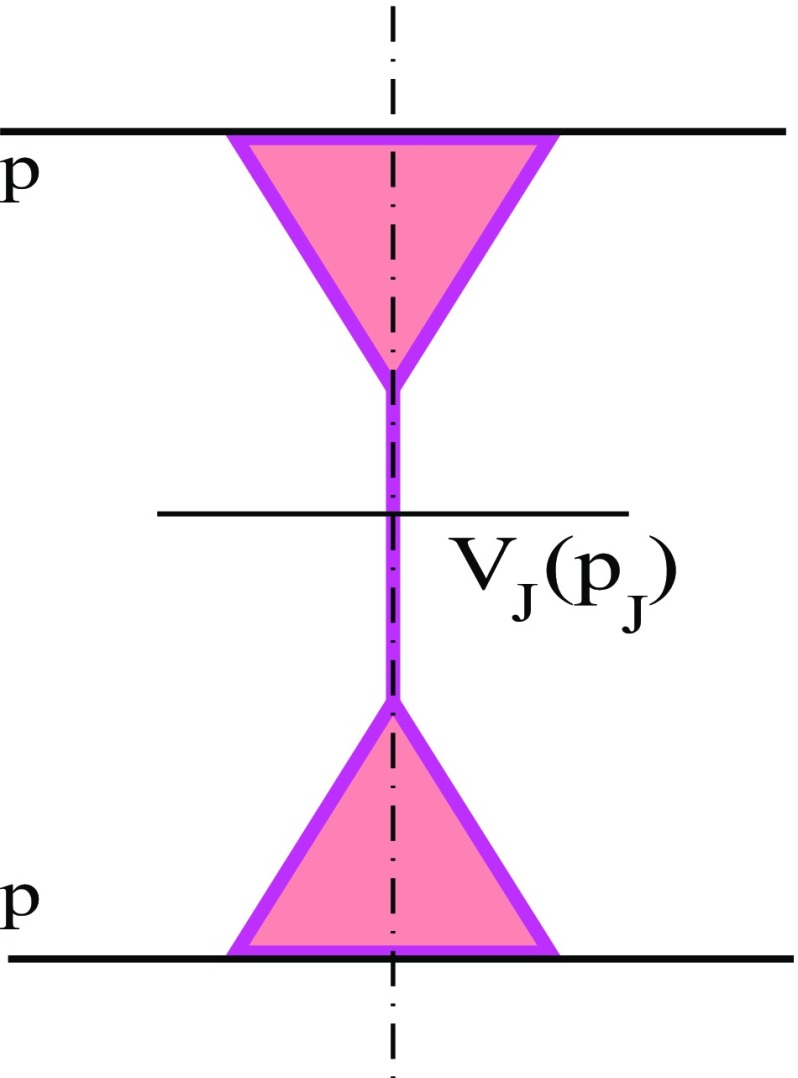
Schematic view for the general RFT diagram for inclusive jet production in *pp* collisions: the projectile and target “triangles” consist of fanlike enhanced Pomeron graphs; $V_{J}(p_{J})$ is the parton *J* emission vertex from a cut Pomeron. The cut plane is shown by the vertical dotted-dashed line

**Fig. 2:**
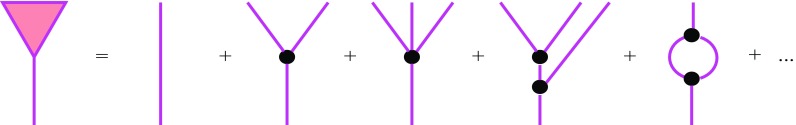
Examples of enhanced Pomeron graphs of lowest orders, contributing to the structure of the projectile and target triangles in Fig. 1; Pomerons are shown by thick lines and multi-Pomeron vertexes by filled circles

To set the scene, let us start with the inclusive cross section for high $p_{\mathrm{t}}$ jet production. Partial contributions to this cross section from various configurations of proton–proton collisions generally involve multiple scattering processes, containing additional soft ($|q^{2}|<Q_{0}^{2}$) and hard ($|q^{2}|>Q_{0}^{2}$) parton cascades fragmenting into secondary hadrons, as well as virtual parton cascades describing elastic rescatterings between constituent partons of the protons. Nevertheless, by virtue of the Abramovskii–Gribov–Kancheli (AGK) cancellations [37], such multiple scattering processes give zero contribution to the inclusive cross section of interest, which is described by Kancheli–Mueller-type diagrams depicted in Fig. 1. The internal structure of the projectile and target triangles in Fig. 1 is explained in Fig. 2: it contains both the basic contribution of an “elementary” parton cascade described as a single Pomeron emission by the parent hadron [1st graph in the right-hand side (rhs) of Fig. 2] and various absorptive corrections to that process due to rescatterings of intermediate partons in the cascade off the parent hadron and off each other.

As a result, we obtain the usual collinear factorization ansatz for the inclusive cross section $\sigma _{pp}^{2\mathrm{jet}}(s,p_{\mathrm{t}}^{\mathrm{cut}})$ for the production of a pair of jets of transverse momentum $p_{\mathrm{t}}>p_{\mathrm{t}}^{\mathrm{cut}}$:

1 $$\begin{aligned}&\sigma _{pp}^{2\mathrm{jet}}(s,p_{\mathrm{t}}^{\mathrm{cut}})\nonumber \\&\quad = \int d^2b \, d^2b' \int dx^{+}\, dx^{-}\int _{p_{\mathrm{t}}>p_{\mathrm{t}}^{\mathrm{cut}}} dp_{\mathrm{t}}^{2} \nonumber \\&\qquad \times \; \sum _{I,J=q,\bar{q},g} \frac{d\sigma _{IJ}^{2\rightarrow 2}(x^{+}x^{-}s,p_{\mathrm{t}}^{2})}{dp_{\mathrm{t}}^{2}}\; G_{I}(x^{+},M_{\mathrm{F}}^{2},b')\nonumber \\&\qquad \times \; G_{J}(x^{-},M_{\mathrm{F}}^{2},|\mathbf {b}-\mathbf {b}'|) \,, \end{aligned}$$

where for future convenience we keep the impact parameter *b* dependence and express the integrand in the rhs of Eq. (1) via the generalized parton distributions (GPDs) in impact parameter space $G_{I}(x,Q^{2},b)$, instead of the usual integrated parton distribution functions (PDFs), $f_{I}(x,Q^{2})=\int d^2b\;G_{I}(x,Q^{2},b)$. Here *s* is the center of mass (c.m.) energy squared, $x^{\pm }$ - parton light-cone momentum fractions, $M_{\mathrm{F}}^{2}$ - the factorization scale, and $d\sigma _{IJ}^{2\rightarrow 2}(\hat{s},p_{t}^{2})/dp_{\mathrm{t}}^{2}$ is the parton scatter cross section.

**Fig. 3:**
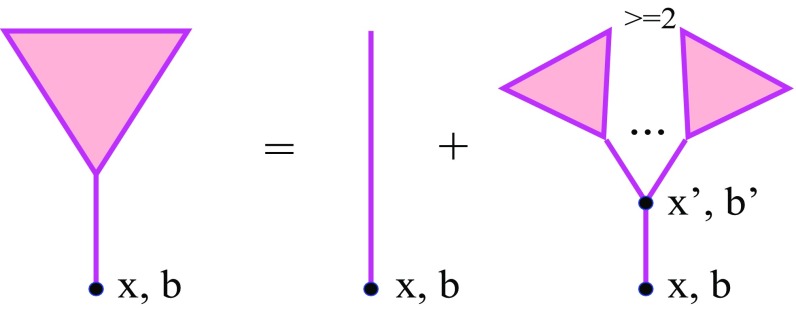
Recursive equation for a fan diagram contribution $\chi _{(i)}^{\mathrm{fan}}(\hat{s},b)$, $\hat{s}=s_{0}/x$

The GPDs for arbitrary $Q^{2}>Q_{0}^{2}$ are obtained evolving the input ones from the cutoff scale $Q_{0}^{2}$:

2 $$\begin{aligned} G_{I}(x,Q^{2},b)= & {} \sum _{I'}\int _{x}^{1}\frac{dz}{z}\, E_{I'\rightarrow I}(z,Q_{0}^{2},Q^{2})\nonumber \\&\times \; G_{I'}(x/z,Q_{0}^{2},b)\,, \end{aligned}$$

with $E_{I\rightarrow J}(z,q^{2},Q^{2})$ being the solution of the DGLAP equations for the initial condition $E_{I\rightarrow J}(z,q^{2},q^{2})=\delta _{I}^{J}\,\delta (1-z)$. In turn, $G_{I}(x,Q_0^{2},b)$ is defined summing over partial contributions of different diffractive eigenstates $|i\rangle$ of the proton, with the partial weights $C_{i}$, as^3^ [30, 38]

3 $$\begin{aligned} x\,G_{I}(x,Q_0^{2},b)= & {} \sum _i C_i \left\{ \chi _{(i)I}^{\mathbb {P}}(s_{0}/x,b) \right. \nonumber \\&+ \;G\int d^{2}b'\int \frac{dx'}{x'} \; \chi _{\mathbb {P}I}^{\mathbb {P}}(s_{0}\,x'/x,|\mathbf {b}-\mathbf {b}'|) \nonumber \\&\times \left. \left[ 1-e^{-\chi _{(i)}^{\mathrm{fan}}(s_{0}/x',b')} -\chi _{(i)}^{\mathrm{fan}}(s_{0}/x',b')\right] \right\} ,\nonumber \\ \end{aligned}$$

being expressed via the solution $\chi _{(i)}^{\mathrm{fan}}$ of the “fan” diagram equation of Fig. 3,

4 $$\begin{aligned} \chi _{(i)}^{\mathrm{fan}}(\hat{s},b)= & {} \chi _{(i)\mathbb {P}}^{\mathbb {P}}(\hat{s},b)\nonumber \\&+\;G\int d^{2}b'\int \frac{dx'}{x'}\; \chi _{\mathbb {PP}}^{\mathbb {P}}(x'\hat{s},|\mathbf {b}-\mathbf {b}'|) \nonumber \\&\times \left[ 1-e^{-\chi _{(i)}^{\mathrm{fan}}(s_{0}/x',b')} -\chi _{(i)}^{\mathrm{fan}}(s_{0}/x',b')\right] . \end{aligned}$$

In Eqs. (3–4), $s_0=1$ GeV$^2$ is the hadronic mass scale and the eikonals $\chi _{(i)\mathbb {P}}^{\mathbb {P}}$ and $\chi _{\mathbb {PP}}^{\mathbb {P}}$ correspond to Pomeron exchanges between the proton diffractive eigenstate $|i\rangle$ and a multi-Pomeron vertex or, respectively, between two multi-Pomeron vertexes, while $\chi _{(i)I}^{\mathbb {P}}$ and $\chi _{\mathbb {P}I}^{\mathbb {P}}$ describe Pomerons coupled to parton *I* on one side and to the proton represented by its diffractive eigenstate $|i\rangle$ or, respectively, to a multi-Pomeron vertex, on the other side, as discussed in more detail in [30, 31]. It is easy to see that the expression in the curly brackets in Eq. (3) is obtained from the rhs of Eq. (4) under the replacements $\chi _{(i)\mathbb {P}}^{\mathbb {P}} \rightarrow \chi _{(i)I}^{\mathbb {P}}$, $\chi _{\mathbb {PP}}^{\mathbb {P}} \rightarrow \chi _{\mathbb {P}I}^{\mathbb {P}}$, i.e. by picking up parton *I* from the downmost Pomeron.

It is noteworthy that Eqs. (2–4) have been derived in Ref. [30], neglecting parton transverse diffusion during the perturbative ($|q^{2}|>Q_{0}^{2}$) evolution and assuming Pomeron–Pomeron interactions to be mediated by nonperturbative parton processes, using the vertexes for the transition of *m* into *n* Pomerons of the form [27]: $G^{(m,n)}=G\,\gamma _{\mathbb {P}}^{m+n}$, where *G* is related to the triple-Pomeron coupling $r_{3\mathbb {P}}$ as $G=r_{3\mathbb {P}}/(4\pi \gamma _{\mathbb {P}}^{3}$). For smaller *x*, the soft ($|q^{2}|<Q_{0}^{2}$) parton evolution proceeds over a longer rapidity interval and results in a larger transverse spread of the parton cloud at the scale $Q_{0}^{2}$, due to the transverse diffusion. On the other hand, for a higher scale $Q^{2}$, a larger part of the available rapidity range is “eaten” by the perturbative evolution [c.f. Eq. (2)]. As a consequence, for a given *x*, partons of higher $Q^{2}$ are distributed over a smaller transverse area.

**Fig. 4:**
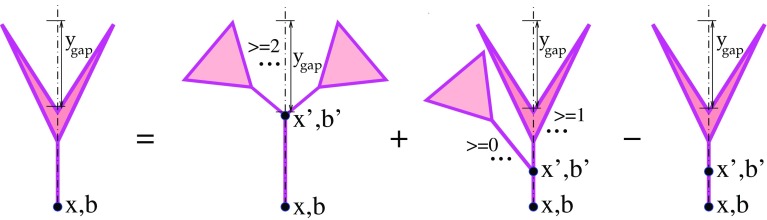
Recursive equation for the contribution $2\chi _{(i)}^{\mathrm{fan(D)}}(\hat{s},b,y^\mathrm{gap})$ of diffractive cuts of fan diagrams of Fig. 3, $\hat{s}=s_{0}/x$. The cut plane is shown by the vertical dotted-dashed lines; the rapidity gaps are also indicated

If we naively assumed the same kind of factorization for diffractive dijet production, the respective cross sections would be defined by subsets of cut diagrams corresponding to Fig. 1, characterized by a desirable structure of rapidity gaps. For example, for the case of central hard diffraction (“double Pomeron exchange”), with the forward and backward rapidity gaps being larger than $y^\mathrm{gap}$, we would obtain

5 $$\begin{aligned}&\sigma _{pp}^{2\mathrm{jet-DPE(fact)}}(s,p_{\mathrm{t}}^{\mathrm{cut}} ,y^\mathrm{gap})\nonumber \\&\quad = \int d^2b \, d^2b' \int dx^{+}dx^{-} \nonumber \\&\qquad \times \int _{p_{\mathrm{t}}>p_{\mathrm{t}}^{\mathrm{cut}}} dp_{\mathrm{t}}^{2} \; \sum _{I,J=q,\bar{q},g} \frac{d\sigma _{IJ}^{2\rightarrow 2}(x^{+}x^{-}s,p_{\mathrm{t}}^{2})}{dp_{\mathrm{t}}^{2}} \nonumber \\&\qquad \times \;G^D_{I}(x^{+},M_{\mathrm{F}}^{2},b',y^\mathrm{gap})\, G^D_{J}(x^{-},M_{\mathrm{F}}^{2},|\mathbf {b}-\mathbf {b}'|,y^\mathrm{gap}).\nonumber \\ \end{aligned}$$

Here the diffractive GPDs $G^D_{I}(x,Q^{2},b,y^\mathrm{gap})$ for an arbitrary scale $Q^{2}$ are obtained via DGLAP evolution from $Q_{0}^{2}$ till $Q^{2}$ [similarly to Eq. (2)], while $G^D_{I}(x,Q_0^{2},b,y^\mathrm{gap})$ is expressed via the contribution $2\chi _{(i)}^{\mathrm{fan(D)}}$ of diffractive cuts of the fan diagrams of Fig. 3 as

6 $$\begin{aligned}&x\,G^D_{I}(x,Q_0^{2},b,y^\mathrm{gap})\nonumber \\&\quad = \sum _i C_i \left\{ \frac{G}{2}\int d^{2}b'\int \frac{dx'}{x'} \right. \nonumber \\&\qquad \times \; \varTheta (-\ln x'-y^\mathrm{gap}) \; \chi _{\mathbb {P}I}^{\mathbb {P}}(s_{0}\, x'/x,|\mathbf {b}-\mathbf {b}'|) \nonumber \\&\qquad \times \left[ (1-e^{-\chi _{(i)}^{\mathrm{fan}}(s_{0}/x',b')})^2 + (e^{2\chi _{(i)}^{\mathrm{fan(D)}}(s_{0}/x',b',y^\mathrm{gap})}-1) \right. \nonumber \\&\qquad \times \left. \left. e^{-2\chi _{(i)}^{\mathrm{fan}}(s_{0}/x',b')} - 2\chi _{(i)}^{\mathrm{fan(D)}}(s_{0}/x',b',y^\mathrm{gap}) \right] \right\} . \end{aligned}$$

The latter are defined by the recursive equation of Fig. 4:

7 $$\begin{aligned}&2\chi _{(i)}^{\mathrm{fan(D)}}(\hat{s},b,y^\mathrm{gap})\nonumber \\&\quad = G\int d^{2}b'\int \frac{dx'}{x'} \nonumber \\&\qquad \times \; \varTheta (-\ln x'-y^\mathrm{gap})\; \chi _{\mathbb {PP}}^{\mathbb {P}}(x'\hat{s},|\mathbf {b}-\mathbf {b}'|) \nonumber \\&\qquad \times \left[ (1-e^{-\chi _{(i)}^{\mathrm{fan}}(s_{0}/x',b')})^2 + (e^{2\chi _{(i)}^{\mathrm{fan(D)}}(s_{0}/x',b',y^\mathrm{gap})}-1) \right. \nonumber \\&\qquad \times \left. e^{-2\chi _{(i)}^{\mathrm{fan}}(s_{0}/x',b')} \; -2\chi _{(i)}^{\mathrm{fan(D)}}(s_{0}/x',b',y^\mathrm{gap}) \right] . \end{aligned}$$

Similarly to Eqs. (3–4), the expression in the curly brackets in Eq. (6) is obtained from the rhs of Eq. (7) under the replacement $\chi _{\mathbb {PP}}^{\mathbb {P}} \rightarrow \chi _{\mathbb {P}I}^{\mathbb {P}}$.

**Fig. 5:**
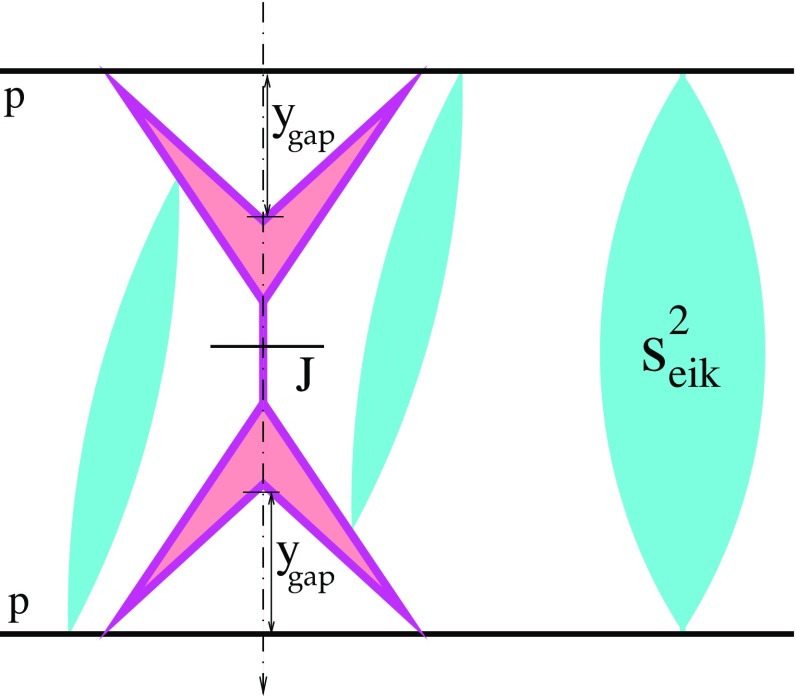
Schematic view for central hard diffraction; parton *J* is emitted from a cut Pomeron at the central rapidity. Eikonal absorption due to constituent parton rescatterings is shown symbolically by the vertical ellipse marked “$S^2_\mathrm{eik}$”; noneikonal absorptive corrections due to rescatterings of intermediate partons mediating the diffractive scattering are indicated by inclined ellipses. The cut plane is shown by the vertical dotted-dashed line; the rapidity gaps are also indicated

The analog of Eq. (5) for single (here, projectile) hard diffraction is^4^

8 $$\begin{aligned}&\sigma _{pp}^{2\mathrm{jet-SD(fact)}}(s,p_{\mathrm{t}}^{\mathrm{cut}} ,y^\mathrm{gap}) \nonumber \\&\quad = \int d^2b \, d^2b'\int dx^{+}dx^{-} \nonumber \\&\qquad \times \int _{p_{\mathrm{t}}>p_{\mathrm{t}}^{\mathrm{cut}}} dp_{\mathrm{t}}^{2} \; \sum _{I,J=q,\bar{q},g} \frac{d\sigma _{IJ}^{2\rightarrow 2}(x^{+}x^{-}s,p_{\mathrm{t}}^{2})}{dp_{\mathrm{t}}^{2}}\nonumber \\&\qquad \times \;G^D_{I}(x^{+},M_{\mathrm{F}}^{2},b',y^\mathrm{gap})\, G_{J}(x^{-},M_{\mathrm{F}}^{2},|\mathbf {b}-\mathbf {b}'|) \,. \end{aligned}$$

Since the integrated diffractive PDFs $f^D_{I}(x,Q^{2},y^\mathrm{gap})= \int d^2b \, G^D_{I}(x,Q^{2},b,y^\mathrm{gap})$ can be inferred from experimental studies of diffractive deep inelastic scattering, Eqs. (5) and (8) could have been well-defined predictions. In reality, there is no good reason to assume such kind of factorization for not fully inclusive quantities, like diffractive cross sections, and the real picture is significantly more complicated, as shown symbolically in Fig. 5.

First, the expressions in the rhs of Eqs. (5) and (8) have to be supplemented by the probability that the desirable rapidity gaps are not filled by secondary particles produced in additional inelastic scatterings processes between constituent partons of the projectile and target protons. For given diffractive eigenstates $|i\rangle$, $|j\rangle$ of the two protons and impact parameter *b*, the corresponding RGS probability is $\exp (-\varOmega _{ij}(s,b))$, where the so-called opacity $\varOmega _{ij}$ is defined as twice the sum over imaginary parts of all significant irreducible Pomeron graphs coupled to the eigenstates $|i\rangle$ and $|j\rangle$.

A relatively compact expression for $\varOmega _{ij}$ has been obtained in [28, 29] summing the contributions of arbitrary Pomeron “nets” exchanged between the projectile and target protons:^5^

9 $$\begin{aligned} \varOmega _{ij}(s,b)= & {} 2\chi _{ij}^{\mathbb {P}}(s,b)+2G\int d^{2}b'\int \frac{dx'}{x'} \left\{ (1\right. \nonumber \\&-\;e^{-\chi _{(i)|(j)}^{\mathrm{net}}(s_{0}/x',\mathbf {b}'|s,\mathbf {b})})\, (1-e^{-\chi _{(j)|(i)}^{\mathrm{net}}(x' s,\mathbf {b}-\mathbf {b}'|s,\mathbf {b})}) \nonumber \\&-\;\chi _{(i)|(j)}^{\mathrm{net}}(s_{0}/x',\mathbf {b}'|s,\mathbf {b}) \, \chi _{(j)|(i)}^{\mathrm{net}}(x' s,\mathbf {b}-\mathbf {b}'|s,\mathbf {b}) \nonumber \\&-\, (\chi _{(i)|(j)}^{\mathrm{net}}(s_{0}/x',\mathbf {b}'|s,\mathbf {b}) -\chi _{(i)\mathbb {P}}^{\mathbb {P}}(s_{0}/x',b')) \nonumber \\&\times \left[ (1-e^{-\chi _{(j)|(i)}^{\mathrm{net}}(x' s,\mathbf {b}-\mathbf {b}'|s,\mathbf {b})})\, e^{-\chi _{(i)|(j)}^{\mathrm{net}}(s_{0}/x',\mathbf {b}'|s,\mathbf {b})} \right. \nonumber \\&- \left. \left. \chi _{(j)|(i)}^{\mathrm{net}}(x' s,\mathbf {b}-\mathbf {b}'|s,\mathbf {b})\right] \right\} , \end{aligned}$$

where $\chi _{ij}^{\mathbb {P}}(s,b)$ is the eikonal for a single Pomeron exchange between the eigenstates $|i\rangle$ and $|j\rangle$ while the “net-fan” eikonal $\chi _{(i)|(j)}^{\mathrm{net}}$ corresponds to the summary contribution of arbitrary irreducible Pomeron nets exchanged between the projectile and target protons (represented by the eigenstates $|i\rangle$ and $|j\rangle$) and coupled to a given multi-Pomeron vertex, which is defined by the recursive equation [c.f. Eq. (4)]:

10 $$\begin{aligned}&\chi _{(i)|(j)}^{\mathrm{net}}(\hat{s},\mathbf {b}''|s,\mathbf {b})\nonumber \\&\quad = \chi _{(i)\mathbb {P}}^{\mathbb {P}}(\hat{s},b'') +G\int d^{2}b'\int \frac{dx'}{x'} \nonumber \\&\qquad \times \; \chi _{\mathbb {PP}}^{\mathbb {P}}(x'\hat{s},|\mathbf {b}''-\mathbf {b}'|) \left[ (1-e^{-\chi _{(i)|(j)}^{\mathrm{net}}(s_{0}/x',\mathbf {b}'|s,\mathbf {b})})\right. \nonumber \\&\qquad \times \left. e^{-\chi _{(j)|(i)}^{\mathrm{net}}(x's,\mathbf {b}-\mathbf {b}'|s,\mathbf {b})} -\chi _{(i)|(j)}^{\mathrm{net}}(s_{0}/x',\mathbf {b}'|s,\mathbf {b})\right] . \end{aligned}$$

Taking into consideration only the above-discussed eikonal rapidity gap suppression, shown symbolically by the vertical ellipse in Fig. 5, Eqs. (5) and (8) will change to

11 $$\begin{aligned}&\sigma _{pp}^{2\mathrm{jet-DPE(eik)}}(s,p_{\mathrm{t}}^{\mathrm{cut}} ,y^\mathrm{gap}) \nonumber \\&\quad = \int d^2b \, d^2b' \int dx^{+}dx^{-} \nonumber \\&\qquad \times \int _{p_{\mathrm{t}}>p_{\mathrm{t}}^{\mathrm{cut}}} dp_{\mathrm{t}}^{2}\; \sum _{I,J=q,\bar{q},g} \frac{d\sigma _{IJ}^{2\rightarrow 2}(x^{+}x^{-}s,p_{\mathrm{t}}^{2})}{dp_{\mathrm{t}}^{2}} \nonumber \\&\qquad \times \; \sum _{i,j} C_i\, C_j \; G^D_{I(i)}(x^{+},M_{\mathrm{F}}^{2},b',y^\mathrm{gap}) \nonumber \\&\qquad \times \; G^D_{J(j)}(x^{-},M_{\mathrm{F}}^{2},|\mathbf {b}-\mathbf {b}'|,y^\mathrm{gap})\; e^{-\varOmega _{ij}(s,b)} \end{aligned}$$

12 $$\begin{aligned}&\sigma _{pp}^{2\mathrm{jet-SD(eik)}}(s,p_{\mathrm{t}}^{\mathrm{cut}} ,y^\mathrm{gap}) \nonumber \\&\quad = \int d^2b \, d^2b' \int dx^{+}dx^{-} \nonumber \\&\qquad \times \int _{p_{\mathrm{t}}>p_{\mathrm{t}}^{\mathrm{cut}}} dp_{\mathrm{t}}^{2}\; \sum _{I,J=q,\bar{q},g} \frac{d\sigma _{IJ}^{2\rightarrow 2}(x^{+}x^{-}s,p_{\mathrm{t}}^{2})}{dp_{\mathrm{t}}^{2}} \nonumber \\&\qquad \times \; \sum _{i,j} C_i\, C_j \; G^D_{I(i)}(x^{+},M_{\mathrm{F}}^{2},b',y^\mathrm{gap}) \nonumber \\&\qquad \times \; G_{J(j)}(x^{-},M_{\mathrm{F}}^{2},|\mathbf {b}-\mathbf {b}'|)\; e^{-\varOmega _{ij}(s,b)} , \end{aligned}$$

where $G_{I(i)}$ and $G^D_{I(i)}$ are obtained evolving from $Q_0^2$ till $M_{\mathrm{F}}^{2}$ the corresponding partial contributions [expressions in the curly brackets in Eqs. (3) and (6), respectively] of the eigenstate $|i\rangle$.

Neglecting color fluctuations in the interacting protons, i.e. considering a single eigenstate $i\equiv 1$, would significantly simplify the analysis since the total opacity $\varOmega _{pp}(s,b)$ can be inferred from measurements of the differential elastic *pp* cross section. Yet, as already stressed previously [3, 11, 13], even in such a case the overall RGS factor would not be a universal constant, depending generally on the process under study and the respective kinematics. In the particular case of diffractive dijet production, considered here, a higher jet transverse momentum cutoff $p_{\mathrm{t}}^{\mathrm{cut}}$ implies a lower probability for the rapidity gap survival, since a larger part of the available rapidity range will be “eaten” by the DGLAP evolution of $G_{I(i)}$ and $G^D_{I(i)}$ in the high $q^2$ range [c.f. Eq. (2)]. Hence, a smaller part will be left for parton transverse diffusion during the soft evolution at $|q^{2}|<Q_{0}^{2}$, with the end result that the contribution of moderately large impact parameters *b* to the integrands of Eqs. (11–12) will be reduced. On the other hand, diffractive production at small *b* is strongly suppressed by a higher opacity $\varOmega _{pp}(s,b)$, which reflects a higher probability for additional inelastic scattering processes, due to a stronger overlap of parton clouds of the interacting protons.

While Eqs. (3) and (6) already account for absorptive corrections to $G_{I}$ and $G^D_{I}$ due to rescatterings of intermediate partons off their parent protons, additional suppression, shown symbolically by the inclined ellipses in Fig. 5, comes from their elastic rescatterings off the partner protons. Intermediate partons in the cascades mediating those rescatterings may in turn scatter elastically off the initial protons, etc. Taking these effects into consideration, we obtain, similarly to the case of soft diffraction in Refs. [24, 30], the cross sections for central and single (here, projectile) diffractive dijet production as

13 $$\begin{aligned}&\sigma _{pp}^{2\mathrm{jet-DPE}}(s,p_{\mathrm{t}}^{\mathrm{cut}} ,y^\mathrm{gap})\nonumber \\&\quad = \int d^2b \, d^2b' \int dx^{+}dx^{-} \nonumber \\&\qquad \times \int _{p_{\mathrm{t}}>p_{\mathrm{t}}^{\mathrm{cut}}} dp_{\mathrm{t}}^{2}\; \sum _{I,J=q,\bar{q},g} \frac{d\sigma _{IJ}^{2\rightarrow 2}(x^{+}x^{-}s,p_{\mathrm{t}}^{2})}{dp_{\mathrm{t}}^{2}} \nonumber \\&\qquad \times \sum _i C_i\, C_j\; \tilde{G}^D_{I(i)|(j)}(x^{+},M_{\mathrm{F}}^{2},\mathbf {b}',y^\mathrm{gap}|s,\mathbf {b}) \nonumber \\&\qquad \times \; \tilde{G}^D_{J(j)|(i)}(x^{-},M_{\mathrm{F}}^{2},\mathbf {b}-\mathbf {b}',y^\mathrm{gap}|s,\mathbf {b})\; e^{-\varOmega _{ij}(s,b)} \end{aligned}$$

14 $$\begin{aligned}&\sigma _{pp}^{2\mathrm{jet-SD}}(s,p_{\mathrm{t}}^{\mathrm{cut}} ,y^\mathrm{gap}) = \int d^2b \, d^2b' \int dx^{+}dx^{-} \nonumber \\&\qquad \times \int _{p_{\mathrm{t}}>p_{\mathrm{t}}^{\mathrm{cut}}} dp_{\mathrm{t}}^{2} \;\sum _{I,J=q,\bar{q},g} \frac{d\sigma _{IJ}^{2\rightarrow 2}(x^{+}x^{-}s,p_{\mathrm{t}}^{2})}{dp_{\mathrm{t}}^{2}} \nonumber \\&\qquad \times \sum _i C_i\, C_j \; \tilde{G}^D_{I(i)|(j)}(x^{+},M_{\mathrm{F}}^{2},\mathbf {b}',y^\mathrm{gap}|s,\mathbf {b}) \nonumber \\&\qquad \times \; \tilde{G}_{J(j)|(i)}(x^{-},M_{\mathrm{F}}^{2},\mathbf {b}-\mathbf {b}'|s,\mathbf {b})\; e^{-\varOmega _{ij}(s,b)} , \end{aligned}$$

where $\tilde{G}_{I(i)|(j)}$ and $\tilde{G}^D_{I(i)|(j)}$ now depend explicitly on the geometry of the *pp* collision, being defined at the $Q_0^{2}$-scale as

15 $$\begin{aligned}&x\,\tilde{G}_{I(i)|(j)}(x,Q_0^{2},\mathbf {b}''|s,\mathbf {b}) \nonumber \\&\quad = \chi _{(i)I}^{\mathbb {P}}(s_{0}/x,b'') \nonumber \\&\qquad +\;G\int d^{2}b'\int \frac{dx'}{x'} \; \chi _{\mathbb {P}I}^{\mathbb {P}}(s_{0}\,x'/x,|\mathbf {b''}-\mathbf {b}'|) \nonumber \\&\qquad \times \left\{ (1-e^{-\chi _{(i)|(j)}^{\mathrm{net}}(s_{0}/x',\mathbf {b}'|s,\mathbf {b})})\, e^{-2\chi _{(j)|(i)}^{\mathrm{net}}(x's,\mathbf {b}-\mathbf {b}'|s,\mathbf {b})}\right. \nonumber \\&\qquad -\left. \chi _{(i)|(j)}^{\mathrm{net}}(s_{0}/x',\mathbf {b}'|s,\mathbf {b})\right\} \end{aligned}$$

16 $$\begin{aligned}&x\,\tilde{G}^D_{I(i)|(j)}(x,Q_0^{2},\mathbf {b}'',y^\mathrm{gap}|s,\mathbf {b}) = \frac{G}{2}\int d^{2}b'\int \frac{dx'}{x'} \nonumber \\&\qquad \times \; \varTheta (-\ln x'-y^\mathrm{gap})\; \chi _{\mathbb {P}I}^{\mathbb {P}}(s_{0}\, x'/x,|\mathbf {b}''-\mathbf {b}'|) \nonumber \\&\qquad \times \left\{ (1- e^{-\chi _{(i)|(j)}^{\mathrm{net}}(s_{0}/x',\mathbf {b}'|s,\mathbf {b})})^2 \right. \nonumber \\&\qquad \times \; e^{-2\chi _{(j)|(i)}^{\mathrm{net}}(x's,\mathbf {b}-\mathbf {b}'|s,\mathbf {b})} + (e^{2\chi _{(i)|(j)}^{\mathrm{net(D)}}(s_{0}/x',\mathbf {b}',y^\mathrm{gap}|s,\mathbf {b})} \nonumber \\&\qquad -\;1) \; e^{-2\chi _{(i)|(j)}^{\mathrm{net}}(s_{0}/x',\mathbf {b}'|s,\mathbf {b}) -2\chi _{(j)|(i)}^{\mathrm{net}}(x's,\mathbf {b}-\mathbf {b}'|s,\mathbf {b})} \nonumber \\&\qquad -\left. 2\chi _{(i)|(j)}^{\mathrm{net(D)}}(s_{0}/x',\mathbf {b}',y^\mathrm{gap}|s,\mathbf {b})\right\} . \end{aligned}$$

**Fig. 6:**
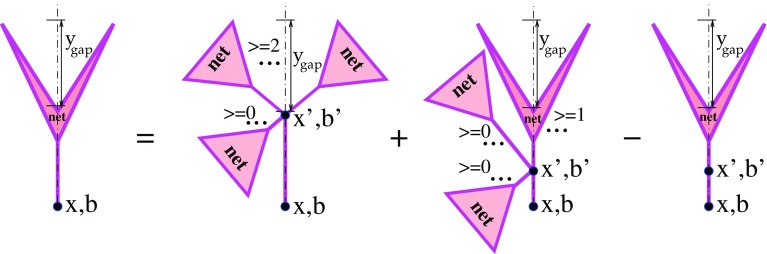
Recursive equation for the contribution $2\chi _{(i)|(j)}^{\mathrm{net(D)}}$ of diffractive cuts of net-fan diagrams. The cut plane is shown by the vertical dotted-dashed line; the rapidity gaps are also indicated

Here the total contribution $2\chi _{(i)|(j)}^{\mathrm{net(D)}}$ of all the unitarity cuts of the net-fan diagrams, characterized by the desirable rapidity gap signature, is defined by the recursive equation of Fig. 6 [c.f. Fig. 4 and Eq. (7)]:

17 $$\begin{aligned}&2\chi _{(i)|(j)}^{\mathrm{net(D)}}(\hat{s},\mathbf {b}'',y^\mathrm{gap}|s,\mathbf {b})\nonumber \\&\quad = G\int d^{2}b'\int \frac{dx'}{x'} \nonumber \\&\qquad \times \; \varTheta (-\ln x'-y^\mathrm{gap})\; \chi _{\mathbb {PP}}^{\mathbb {P}}(x'\hat{s},|\mathbf {b}''-\mathbf {b}'|) \nonumber \\&\qquad \times \left\{ (1-e^{-\chi _{(i)|(j)}^{\mathrm{net}}(s_{0}/x',\mathbf {b}'|s,\mathbf {b})})^2 e^{-2\chi _{(j)|(i)}^{\mathrm{net}}(x's,\mathbf {b}-\mathbf {b}'|s,\mathbf {b})}\right. \nonumber \\&\qquad +(e^{2\chi _{(i)|(j)}^{\mathrm{net(D)}}(s_{0}/x',\mathbf {b}',y^\mathrm{gap}|s,\mathbf {b})} -1) \nonumber \\&\qquad \times \; e^{-2\chi _{(i)|(j)}^{\mathrm{net}}(s_{0}/x',\mathbf {b}'|s,\mathbf {b}) -2\chi _{(j)|(i)}^{\mathrm{net}}(x's,\mathbf {b}-\mathbf {b}'|s,\mathbf {b})} \nonumber \\&\qquad -\left. 2\chi _{(i)|(j)}^{\mathrm{net(D)}}(s_{0}/x',\mathbf {b}',y^\mathrm{gap}|s,\mathbf {b})\right\} . \end{aligned}$$

Clearly, the rhs of Eq. (16) is obtained from the rhs of Eq. (17) under the replacement $\chi _{\mathbb {PP}}^{\mathbb {P}} \rightarrow \chi _{\mathbb {P}I}^{\mathbb {P}}$.

In the next section, we apply Eqs. (5), (8), (11–12), and (13–14) to investigate the rapidity gap survival for diffractive dijet production in *pp* collisions. We shall use the parameter set of the QGSJET-II-04 model [31], which has been obtained by fitting the model to available accelerator data on total and elastic proton–proton cross sections, elastic scattering slope, and total and diffractive structure functions $F_{2}$, $F_{2}^{\mathrm{D}(3)}$.

**Fig. 7:**
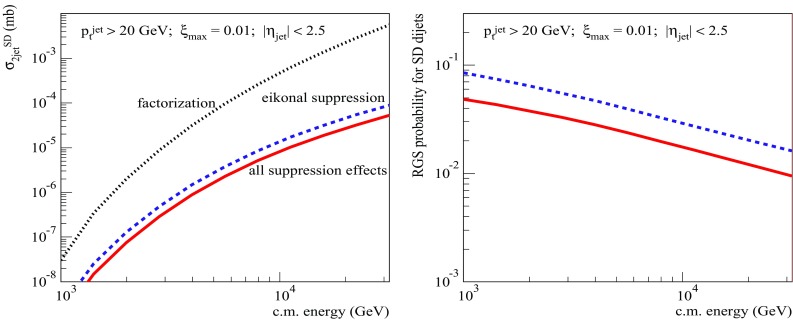
Left: energy dependence of the calculated single diffractive dijet cross sections: $\sigma _{pp}^{2\mathrm{jet-SD(fact)}}$ (dotted), $\sigma _{pp}^{2\mathrm{jet-SD(eik)}}$ (dashed), and $\sigma _{pp}^{2\mathrm{jet-SD}}$ (solid). Right: energy dependence of the corresponding RGS probabilities: $S^2_\mathrm{SD(eik)}$ (dashed) and $S^2_\mathrm{SD(tot)}$ (solid). All for $p_{\mathrm{t}}^{\mathrm{cut}}=20$ GeV/c, $\xi _{\max }=0.01$, and $|\eta _\mathrm{jet}|<2.5$

## Results and discussion

Let us start with the investigation of the energy dependence of the dijet production cross section and of the respective rapidity gap survival probability for single diffractive (SD) proton–proton collisions. In Fig. 7 (left), we compare our results for $\sqrt{s}$-dependence of $\sigma _{pp}^{2\mathrm{jet-SD(fact)}}$ calculated according to Eq. (8), based on the factorization assumption, to the one of $\sigma _{pp}^{2\mathrm{jet-SD(eik)}}$ [Eq. (12)], which accounts for the eikonal rapidity gap suppression, and to $\sigma _{pp}^{2\mathrm{jet-SD}}$ [Eq. (14)], which takes into account all the above-discussed suppression effects. We impose here cuts on the jet transverse momentum, $p_{\mathrm{t}}^{\mathrm{jet}}>p_{\mathrm{t}}^{\mathrm{cut}}=20$ GeV/c, and on the light cone momentum loss by the projectile proton, $\xi =M_X^2/s <\xi _{\max }=0.01$, i.e. $y^\mathrm{gap}=-\ln \xi _{\max }$, with $M_X^2$ being the mass squared of the produced diffractive system. Additionally, we demand both jets to be produced in the central pseudorapidity $\eta$ region, $|\eta _\mathrm{jet}|<2.5$. In Fig. 7 (right), we plot the corresponding RGS factors $S^2_\mathrm{SD(eik)}\equiv \sigma _{pp}^{2\mathrm{jet-SD(eik)}} /\sigma _{pp}^{2\mathrm{jet-SD(fact)}}$ and $S^2_\mathrm{SD(tot)}\equiv \sigma _{pp}^{2\mathrm{jet-SD}} /\sigma _{pp}^{2\mathrm{jet-SD(fact)}}$. While the plotted diffractive dijet cross sections steeply rise with energy, due to the increase of the kinematic space for parton evolution, we observe a mild energy-dependence for the respective RGS factors. Naturally, the probability for the rapidity gap survival goes down at higher energies – due to the increase of parton densities, resulting in an enhancement of multiple scattering, hence, in a decrease of $S^2_\mathrm{SD(eik)}$. However, the additional RG suppression by absorptive corrections of non-eikonal type, reflected by the ratio $S^2_\mathrm{SD(tot)}/S^2_\mathrm{SD(eik)}\simeq 0.6$, appears to be a much weaker and almost energy-independent effect. At the first sight, this seems surprising as the energy rise of parton densities should lead to an enhancement of rescatterings of intermediate partons from the cascades mediating the diffractive scattering, hence, to stronger non-eikonal absorptive corrections.

**Fig. 8:**
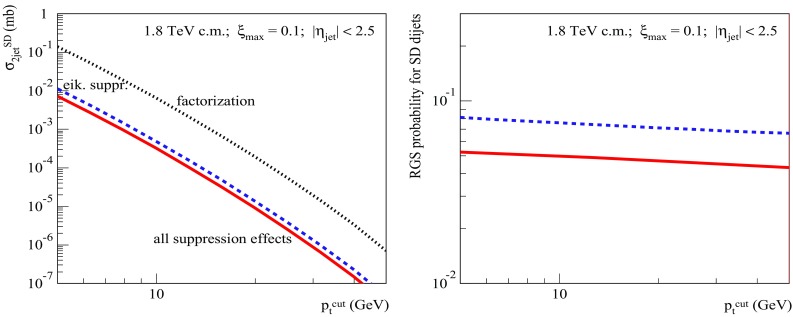
Left: $p_{\mathrm{t}}^{\mathrm{cut}}$-dependence of the calculated SD dijet cross sections: $\sigma _{pp}^{2\mathrm{jet-SD(fact)}}$ (dotted), $\sigma _{pp}^{2\mathrm{jet-SD(eik)}}$ (dashed), and $\sigma _{pp}^{2\mathrm{jet-SD}}$ (solid). Right: $p_{\mathrm{t}}^{\mathrm{cut}}$-dependence of the corresponding RGS probabilities: $S^2_\mathrm{SD(eik)}$ (dashed) and $S^2_\mathrm{SD(tot)}$ (solid). All for $\sqrt{s}=1.8$ TeV, $\xi _{\max }=0.1$, and $|\eta _\mathrm{jet}|<2.5$

**Fig. 9:**
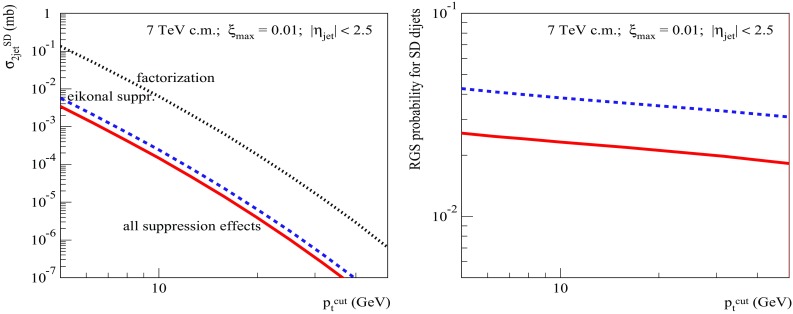
Same as in Fig. 8 for $\sqrt{s}=7$ TeV and $\xi _{\max }=0.01$

To get a further insight into the problem, let us check the dependence of the dijet SD cross sections and of the RGS factors on the jet transverse momentum cutoff $p_{\mathrm{t}}^{\mathrm{cut}}$ at the energies of the Tevatron (Fig. 8), for $\xi _{\max }=0.1$, and the LHC (Fig. 9), for $\xi _{\max }=0.01$. The obtained $p_{\mathrm{t}}^{\mathrm{cut}}$-dependencies for both $S^2_\mathrm{SD(eik)}$ and $S^2_\mathrm{SD(tot)}$ are rather flat. There is a mild decrease of $S^2_\mathrm{SD(eik)}$ for increasing $p_{\mathrm{t}}^{\mathrm{cut}}$ - due to the shift of the dijet production into more opaque region of smaller impact parameters, which is related to the reduction of the phase space available for soft ($|q^{2}|<Q_{0}^{2}$) parton evolution, as discussed in Sect. 2. On the other hand, the ratio $S^2_\mathrm{SD(tot)}/S^2_\mathrm{SD(eik)}$ remains nearly constant over the studied range 5 GeV/c$<p_{\mathrm{t}}^{\mathrm{cut}}<50$ GeV/c. To some extent, this is less surprising than the flat energy-dependence in Fig. 7 (right), since there are two competing effects here, both arising from the reduced kinematic phase space for the soft parton evolution. On the one side, the shift of the dijet production towards smaller impact parameters should enhance the absorptive effects related to rescatterings of intermediate partons. On the other hand, due to the reduction of the rapidity space for the soft parton evolution, one may expect a weakening of those effects.^6^

**Fig. 10:**
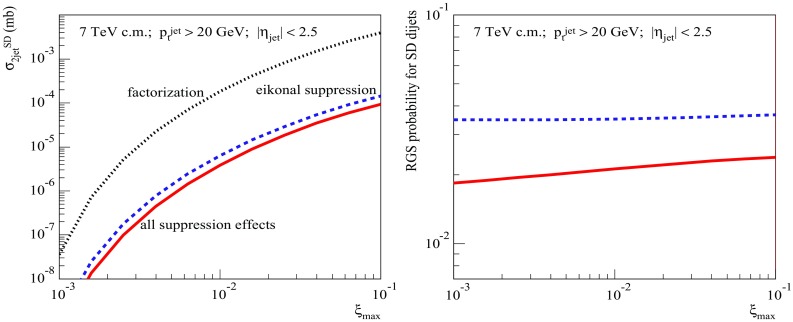
Left: $\xi _{\max }$-dependence of the calculated SD dijet cross sections: $\sigma _{pp}^{2\mathrm{jet-SD(fact)}}$ (dotted), $\sigma _{pp}^{2\mathrm{jet-SD(eik)}}$ (dashed), and $\sigma _{pp}^{2\mathrm{jet-SD}}$ (solid). Right: $\xi _{\max }$-dependence of the corresponding RGS probabilities: $S^2_\mathrm{SD(eik)}$ (dashed) and $S^2_\mathrm{SD(tot)}$ (solid). All for $\sqrt{s}=7$ TeV, $p_{\mathrm{t}}^{\mathrm{cut}}=20$ GeV/c, and $|\eta _\mathrm{jet}|<2.5$

For completeness, let us also study the dependencies of the dijet SD cross sections and of the RGS factors on the size of the rapidity gap. These are plotted in Fig. 10 as a function of $\xi _{\max }$, for the production of jets of $p_{\mathrm{t}}^{\mathrm{jet}}> 20$ GeV/c at the LHC energy $\sqrt{s}=7$ TeV. Here we observe a rather flat behavior for $S^2_\mathrm{SD(eik)}$, since the size of the rapidity gap makes a small impact on the slope for the diffractive scattering, hence, on the range of impact parameters relevant for SD dijet production. On the other hand, for decreasing $\xi _{\max }$ (thus, for an increasing rapidity range for virtual parton cascades mediating the diffractive scattering), there is some enhancement of absorptive effects related to rescatterings of intermediate partons, which results in a slight decrease of the RGS probability, with $S^2_\mathrm{SD(tot)}/S^2_\mathrm{SD(eik)}$ changing from 0.65 for $\xi _{\max }=0.1$ to 0.53 for $\xi _{\max }=10^{-3}$.

**Fig. 11:**
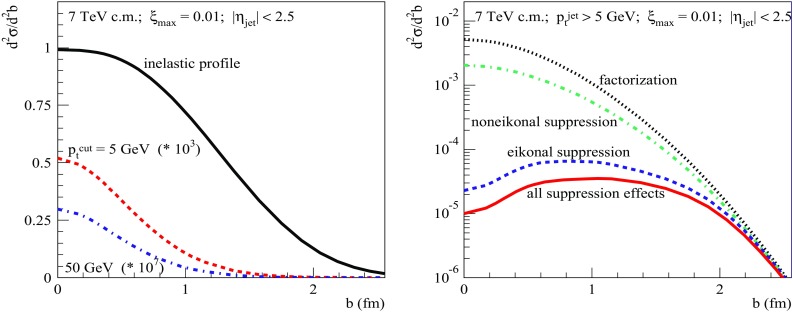
Left: transverse profiles for SD dijet production $d^2\sigma _{pp}^{2\mathrm{jet-SD(fact)}}/d^2b$, calculated based on the factorization assumption, for $p_{\mathrm{t}}^{\mathrm{cut}}=5$ and 50 GeV/c (dashed and dotted-dashed lines, respectively), compared to the inelastic profile (solid line). Right: transverse profiles for SD dijet production for $p_{\mathrm{t}}^{\mathrm{cut}}=5$ GeV/c, calculated taking different absorptive effects into account; dotted, dotted-dashed, dashed, and solid lines correspond, respectively, to $d^2\sigma _{pp}^{2\mathrm{jet-SD(fact)}}/d^2b$, $d^2\sigma _{pp}^{2\mathrm{jet-SD(noneik)}}/d^2b$, $d^2\sigma _{pp}^{2\mathrm{jet-SD(eik)}}/d^2b$, and $d^2\sigma _{pp}^{2\mathrm{jet-SD}}/d^2b$. All for $\sqrt{s}=7$ TeV, $\xi _{\max }=0.01$, and $|\eta _\mathrm{jet}|<2.5$

It is clear from our discussion so far that the key to the understanding of the rapidity gap suppression of diffractive dijet production is in the impact parameter dependence of the respective transverse profiles $d^2\sigma _{pp}^{2\mathrm{jet-SD(fact)}}/d^2b$, $d^2\sigma _{pp}^{2\mathrm{jet-SD(eik)}}/d^2b$, and $d^2\sigma _{pp}^{2\mathrm{jet-SD}}/d^2b$, which are defined by the *b*-integrands of Eqs. (8), (12), and (14), respectively. In Fig. 11 (left), we plot $d^2\sigma _{pp}^{2\mathrm{jet-SD(fact)}}/d^2b$ for $\sqrt{s}=7$ TeV and $\xi _{\max }=10^{-2}$, for two values of the jet $p_{\mathrm{t}}$-cutoff: $p_{\mathrm{t}}^{\mathrm{cut}}=5$ and 50 GeV/c, in comparison to the inelastic profile $G_{pp}^\mathrm{inel}(s,b)=1-\sum _{i,j}C_i\, C_j\; e^{-\varOmega _{ij}(s,b)}$. Here we immediately see the origin of the factorization breaking for diffractive dijet production: the respective profile defined by the factorization ansatz, Eq. (8), is confined to the opaque region of small impact parameter *b*, where the probability of additional inelastic rescatterings between the protons’ constituents is close to unity. When taking into account the eikonal RG suppression, i.e. including the probability for no such rescatterings [factor $e^{-\varOmega _{ij}(s,b)}$ in the rhs of Eq. (12)], the corresponding production rate at $b\simeq 0$ is reduced by many orders of magnitude [c.f. dotted and dashed lines in Fig. 11 (right)]. At $b> 2$ fm, the absorption becomes weak, yet the production rate is miserable there. Hence, the bulk of the SD dijet production comes from the intermediate region $b\sim$ 1–1.5 fm. It is worth stressing that the above-discussed transverse picture is of generic character, being a consequence of the fundamental feature of hadronic collisions, namely, that the slope for diffractive scattering is considerably smaller than the elastic scattering slope $B_{pp}^\mathrm{el}$ which defines the transverse spread of the inelastic profile $G_{pp}^\mathrm{inel}(s,b)$.

Let us next consider the profile $d^2\sigma _{pp}^{2\mathrm{jet-SD(noneik)}}/d^2b$, plotted as the dotted-dashed line in Fig. 11 (right), which corresponds to taking noneikonal absorption into account, while neglecting the eikonal RG suppression. It is defined by the *b*-integrand of Eq. (14), omitting the factors $e^{-\varOmega _{ij}(s,b)}$. We see that the respective effects, being reflected by the differences between the dotted and dotted-dashed lines in the Figure, are strongest at small *b*, i.e. where rescatterings of intermediate partons are enhanced by a higher parton density. However, as discussed above, the contribution of the small *b* region to the diffractive dijet production is strongly suppressed by the eikonal absorption. This explains the relatively weak effect of the noneikonal absorption, observed in Figs. 7, 8, 9 and 10. In other words, as argued in Ref. [1, 14], the eikonal RG suppression effectively eliminates the kinematic region where noneikonal absorptive effects could be of significant importance. This also helps us to understand the very week energy-dependence of the noneikonal absorption, observed in Fig. 7 (right). Moving to higher energies, diffractive dijet production at relatively small *b* is stronger and stronger suppressed by the eikonal RG suppression and important contributions come only from larger impact parameters where the noneikonal absorption becomes weaker.

**Fig. 12:**
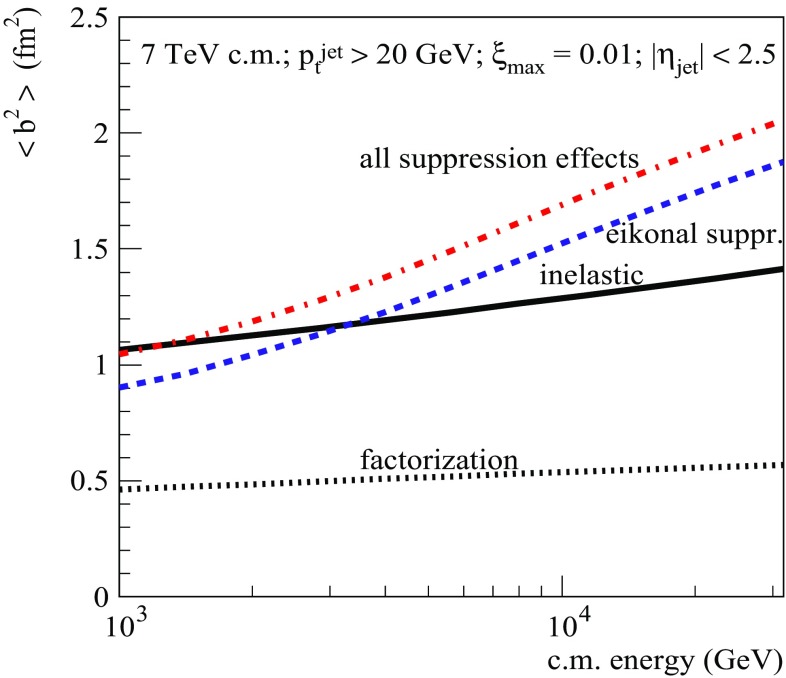
Average $b^2$ for inelastic *pp* collisions (solid) and for SD dijet production for $\sqrt{s}=7$ TeV, $p_{\mathrm{t}}^{\mathrm{cut}}=20$ GeV/c, $\xi _{\max }=0.01$, and $|\eta _\mathrm{jet}|<2.5$, as calculated using the different approximations: factorization (dotted), eikonal suppression (dashed), and all suppression effects (dotted-dashed)

The above-discussed tendencies become more clear if we compare the energy-dependence of the average impact parameter squared for the different approximations, $\langle b_{(X)}^2\rangle =\int d^2b\;b^2\, \frac{d^2\sigma _{pp}^{(X)}}{d^2b}/\sigma _{pp}^{(X)}$ [$X=$ 2jet-SD(fact), 2jet-SD(eik), and 2jet-SD], to the one for general inelastic collisions, $\langle b_\mathrm{inel}^2\rangle =\int d^2b\;b^2\, G_{pp}^\mathrm{inel}(s,b)/\sigma _{pp}^\mathrm{inel}(s)$, as plotted in Fig. 12. We notice that $\langle b_\mathrm{(2jet-SD(fact))}^2\rangle$ calculated based on the factorization assumption is more than twice smaller than $\langle b_\mathrm{inel}^2\rangle$. This is not surprising since, firstly, already the slope for soft diffraction is considerably smaller than $B_{pp}^\mathrm{el}$ and, secondly, in case of hard diffraction a large part of the available rapidity range is “eaten” by hard ($|q^{2}|>Q_{0}^{2}$) parton evolution characterized by weak transverse diffusion, $\varDelta b^2 \sim 1/|q^{2}|$, neglected here. Let us also remark that for increasing energy $\langle b_\mathrm{(2jet-SD(fact))}^2\rangle$ rises slower than $\langle b_\mathrm{inel}^2\rangle$ because the hard parton evolution covers a longer and longer rapidity interval. Next, we notice that, firstly, $\langle b_\mathrm{(2jet-SD(eik))}^2\rangle$ is considerably larger than $\langle b_\mathrm{(2jet-SD(fact))}^2\rangle$ and, secondly, it has a significantly steeper energy rise. This is because the region of small *b* is strongly suppressed by the eikonal absorption [c.f. dotted and dashed lines in Fig. 11 (right)] and for higher energies the strong absorption extends towards larger impact parameters, as a consequence of the widening and “blackening” of the inelastic profile, as noticed already in Ref. [5], which are caused by parton transverse diffusion and the energy rise of parton densities, respectively. The same arguments apply to the noneikonal absorption, due to its dependence on parton density: it is strongest at small *b* and, for increasing energy, becomes more important at larger impact parameters. Consequently, $\langle b_\mathrm{(2jet-SD)}^2\rangle$ is slightly larger than $\langle b_\mathrm{(2jet-SD(eik))}^2\rangle$.

**Fig. 13:**
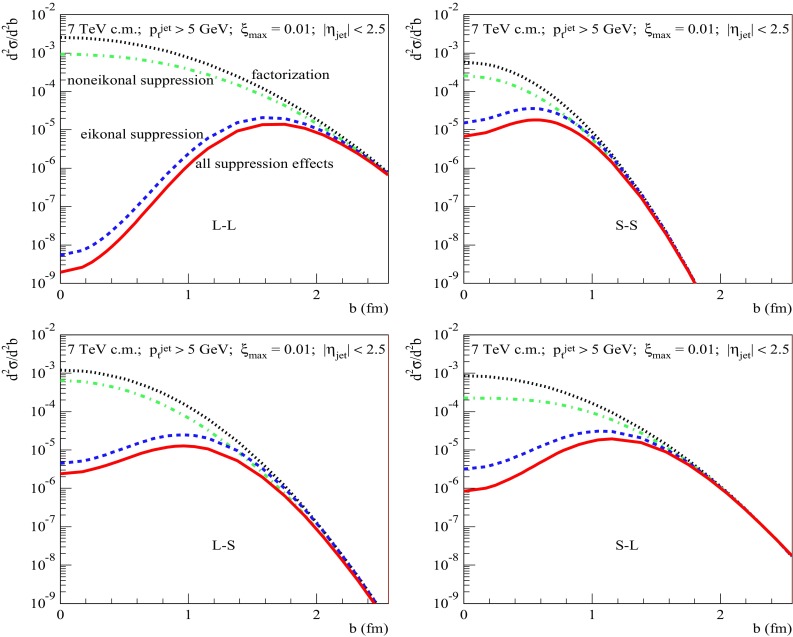
Same as in Fig. 11 (right) for different combinations of diffractive eigenstates of the projectile and target protons, as explained in the text

As the cross section formulas, Eqs. (8), (12), and (14), are obtained averaging over contributions of different Fock states of the projectile and target protons, it may be sensible to discuss the relative roles of the different absorptive corrections separately for particular combinations of those Fock states. This is illustrated in Fig. 13, where we plot for $\sqrt{s}=7$ TeV, $p_{\mathrm{t}}^{\mathrm{cut}}=5$ GeV/c, and $\xi _{\max }=10^{-2}$ the respective partial contributions $d^2\sigma _{pp(ij)}^{2\mathrm{jet-SD(fact)}}/d^2b$, $d^2\sigma _{pp(ij)}^{2\mathrm{jet-SD(noneik)}}/d^2b$, $d^2\sigma _{pp(ij)}^{2\mathrm{jet-SD(eik)}}/d^2b$, and $d^2\sigma _{pp(ij)}^{2\mathrm{jet-SD}}/d^2b$ for the 4 different cases: when both the projectile and the target protons are represented by their largest size Fock states, marked as “L–L” in the Figure, for an interaction between the small size states (“S–S”), and for interactions between Fock states of different sizes (“L–S” and “S–L”). In the “L–L” case, we see that all the above-discussed tendencies, in particular, the strong suppression of the small *b* region are much more prominent, being enhanced by the larger (integrated) parton densities for the large size states. On the other hand, in the “S–S” case, the interaction profile is more transparent due to smaller parton densities, resulting in a weaker absorption at small impact parameters. However, because of the smaller scattering slope, the dijet production is confined here to the small *b* region, thus making a small contribution to the overall yield. A similar competition between the transverse spread and the strength of absorption we observe for the two non-diagonal cases. In the “L–S” case, the diffractive scattering of the projectile proton is enhanced by its larger parton density. Moreover, both the eikonal absorption and the one due to intermediate parton rescatterings off the target proton are reduced because of the lower parton density for the latter. However, the scattering slope in this case is sizably smaller tahn in the “S–L” case. Consequently, the latter contribution appears to be a more important one, despite stronger noneikonal absorptive corrections related to rescatterings of intermediate partons off the target proton which has a higher parton density than in the “L–S” case (c.f. dotted and dotted-dashed lines in the lower right panel of Fig. 13).

**Fig. 14:**
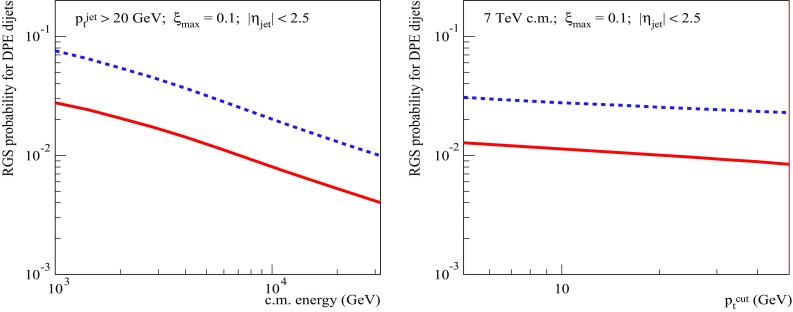
Energy dependence for $p_{\mathrm{t}}^{\mathrm{cut}}= 20$ GeV/c (left) and $p_{\mathrm{t}}^{\mathrm{cut}}$-dependence for $\sqrt{s}=7$ TeV (right) of the RGS factors $S^2_\mathrm{DPE(eik)}$ (dashed) and $S^2_\mathrm{DPE(tot)}$ (solid) for central diffractive dijet production; $\xi _{\max }=0.1$, $|\eta _\mathrm{jet}|<2.5$

**Fig. 15:**
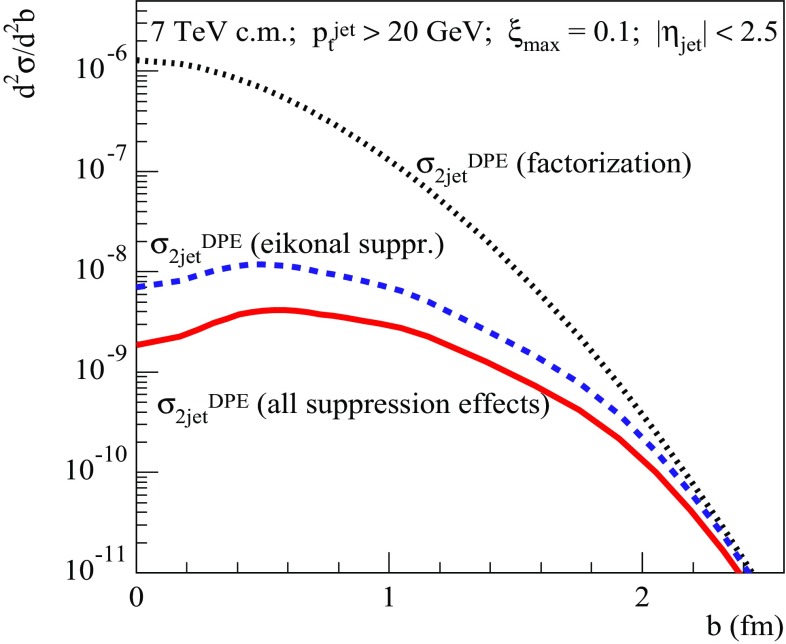
Transverse profiles for central diffractive dijet production at $\sqrt{s}=7$ TeV for $p_{\mathrm{t}}^{\mathrm{jet}}>20$ GeV/c, calculated taking different absorptive effects into account: $d^2\sigma _{pp}^{2\mathrm{jet-SD(fact)}}/d^2b$ (dotted), $d^2\sigma _{pp}^{2\mathrm{jet-SD(eik)}}/d^2b$ (dashed), and $d^2\sigma _{pp}^{2\mathrm{jet-SD}}/d^2b$ (solid); $\xi _{\max }=0.1$, $|\eta _\mathrm{jet}|<2.5$

Let us now turn to the case of central diffractive dijet production. In Fig. 14, we plot the energy (for $p_{\mathrm{t}}^{\mathrm{jet}}> 20$ GeV/c) and the $p_{\mathrm{t}}^{\mathrm{cut}}$ (for $\sqrt{s}=7$ TeV) dependencies of the corresponding RGS factors (all for $\xi _{\max }=0.1$), $S^2_\mathrm{DPE(eik)}= \sigma _{pp}^{2\mathrm{jet-DPE(eik)}} /\sigma _{pp}^{2\mathrm{jet-DPE(fact)}}$ and $S^2_\mathrm{DPE(tot)}= \sigma _{pp}^{2\mathrm{jet-DPE}} /\sigma _{pp}^{2\mathrm{jet-DPE(fact)}}$, where $\sigma _{pp}^{2\mathrm{jet-DPE(fact)}}$, $\sigma _{pp}^{2\mathrm{jet-DPE(eik)}}$, and $\sigma _{pp}^{2\mathrm{jet-DPE}}$ are defined by Eqs. (5), (11), and (13), respectively. Additionally, in Fig. 15 we show the corresponding transverse profiles $d^2\sigma _{pp}^{2\mathrm{jet-DPE(fact)}}/d^2b$, $d^2\sigma _{pp}^{2\mathrm{jet-DPE(eik)}}/d^2b$, and $d^2\sigma _{pp}^{2\mathrm{jet-DPE}}/d^2b$, given by the *b*-integrands in the rhs of Eqs. (5), (11), and (13). Here we observe for the RGS probability the same tendencies as in the case of single diffraction: a relatively weak dependence on the collision energy and a low sensitivity to the jet transverse momentum cutoff, also a nearly constant ratio $S^2_\mathrm{DPE(tot)}/S^2_\mathrm{DPE(eik)}\simeq 0.4$. The overall absorption is approximately twice stronger, compared to SD dijet production, because of the smaller scattering slope for central diffraction, which thus concentrates at smaller impact parameters [c.f. dotted lines in Figs. 11 (right) and 15]. There, both eikonal and noneikonal absorptive corrections are enhanced by the higher parton densities in the projectile and target protons, resulting in a substantial suppression of the production profile, as one can see in Fig.  15. Interestingly, the obtained additional suppression of the RGS probability by noneikonal absorptive effects, $S^2_\mathrm{DPE(eik)}/S^2_\mathrm{DPE(tot)}\simeq 2.5$, fits well in the range [2–3] estimated earlier in Ref. [17] using a different framework.

**Fig. 16:**
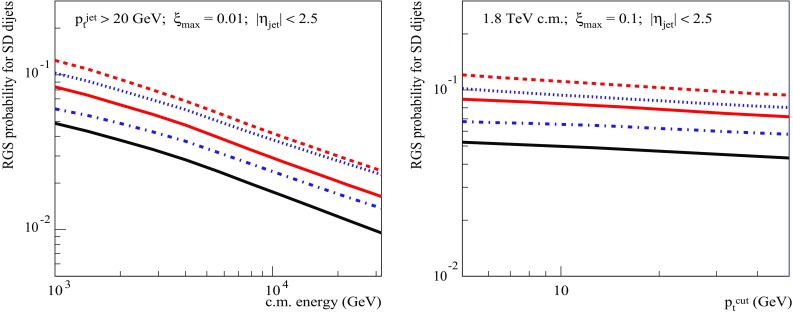
Energy dependence for $p_{\mathrm{t}}^{\mathrm{cut}}= 20$ GeV/c, $\xi _{\max }=0.01$ (left) and $p_{\mathrm{t}}^{\mathrm{cut}}$-dependence for $\sqrt{s}=1.8$ TeV, $\xi _{\max }=0.1$ (right) of the RGS factors $S^2_\mathrm{SD(eik)}$ and $S^2_\mathrm{SD(tot)}$ for SD dijet production, using alternative parameter tunes: SD- (respectively, dashed and upper solid lines), and SD+ (respectively, dotted and dotted-dashed lines); $|\eta _\mathrm{jet}|<2.5$. The downmost solid lines correspond to $S^2_\mathrm{SD(tot)}$ calculated with the default parameters

It may be interesting to check how robust are the presented results with respect to variations of parameters of the adopted model. To address that, we repeat the calculations of the RGS probability for SD dijet production using two alternative parameter tunes of the QGSJET-II-04 model, discussed in Ref. [39] in relation to present uncertainties of soft diffraction measurements at the LHC. One of the tunes, referred to as “SD-”, yields 30% smaller low mass diffraction cross section, compared to the default parameter settings, because of a smaller difference between the strengths of the Pomeron coupling to different diffractive eigenstates of the proton. At parton level, this would correspond to weaker color fluctuations in the proton. The other one, referred to as “SD+”, is characterized by an increased rate of high mass diffraction in *pp* collisions, which has been achieved by using a higher value for the triple-Pomeron coupling, and a slightly smaller low mass diffraction. Apart from those features, both parameter tunes have been calibrated with the same set of experimental data on hadronic cross sections and particle production as the default model (see Ref. [39] for more details). The calculated energy (for $p_{\mathrm{t}}^{\mathrm{cut}}= 20$ GeV/c and $\xi _{\max }=0.01$) and $p_{\mathrm{t}}^{\mathrm{cut}}$ (for $\sqrt{s}=1.8$ TeV and $\xi _{\max }=0.1$) dependencies of the corresponding RGS factors for SD dijet production are shown in Fig. 16, being very similar to each other and to the above-discussed results obtained using the default model parameters. This applies also to the relative importance of the noneikonal absorption: the calculated $S^2_\mathrm{SD(tot)}/S^2_\mathrm{SD(eik)}$ ranges between 0.6 and 0.8, depending on the parameter set and the event selection. However, the absolute value of the RGS probability appears to be quite sensitive to the treatment of low mass diffraction, being some 70% higher for the SD- tune, due to a slightly more transparent inelastic profile, compared to the default case.

Let us finally check whether the obtained values for the RGS probability are compatible with available experimental data. Here the situation is somewhat confusing. At $\sqrt{s}=1.8$ TeV, using the parameter sets of QGSJET-II-04, SD+, and SD- tunes, for SD dijet production ($p_{\mathrm{t}}^{\mathrm{cut}}= 7$ GeV/c and $\xi _{\max }=0.1$) we obtain the values $S^2_\mathrm{SD(tot)}\simeq 0.05$, 0.07, and 0.09, respectively [see Fig. 16 (right)], which are all compatible with the CDF result, $0.06 \pm 0.02$ [40]. However, recent measurements at the LHC by the CMS [41] and ATLAS [42] experiments indicate that the RGS probability for SD dijet production at $\sqrt{s}=7$ TeV ($p_{\mathrm{t}}^{\mathrm{cut}}= 20$ GeV/c and $\xi _{\max }\sim 10^{-3}$) is at 10% level, which is compatible with the CDF result at $\sqrt{s}=1.8$ TeV and is almost an order of magnitude higher than what we obtain here [c.f. Fig. 16 (left)]. Thus, we find the experimental situation very puzzling since the decrease with energy of the RGS probability is closely related to the significant shrinkage of the diffractive cone, convincingly demonstrated by the TOTEM [43–47] and ATLAS [48, 49] measurements. As the scattering slope for (unabsorbed) diffractive dijet production rises with energy slower than $B_{pp}^\mathrm{el}$ (c.f. dotted and solid lines in Fig. 12 for the respective $\langle b^2 \rangle$), at higher energies the bulk of the production becomes confined to more and more opaque region. If the flat energy behavior of the RGS probability is further confirmed, notably, by using also information from proton tagging by forward detectors at the LHC, this would imply a very nontrivial dynamics of hadronic collisions.

## Conclusions

In this work, we applied the phenomenological Reggeon field theory framework for calculations of the rapidity gap survival probability for diffractive dijet production in proton–proton collisions, investigating in some detail various absorptive effects contributing to the RG suppression. Most importantly, we have demonstrated that the absorption due to elastic rescatterings of intermediate partons mediating the diffractive scattering plays a subdominant role, compared to the eikonal rapidity gap suppression due to elastic rescatterings of constituent partons of the colliding protons. The corresponding suppression factors, $S^2_\mathrm{SD(tot)}/S^2_\mathrm{SD(eik)}$ and $S^2_\mathrm{DPE(tot)}/S^2_\mathrm{DPE(eik)}$, are found to depend very weakly on the collision energy and the event kinematics. This is good news since, for a given process of interest, one may account for such effects, in the first crude approximation, via a rescaling of jet rates by a constant factor. On the other hand, such a weak dependence is somewhat accidental, as it results from a complex interplay between particular event selections (e.g. the choice for the jet $p_\mathrm{t}$ cutoff) and the corresponding modification of the transverse profile for diffractive dijet production. Generally, such corrections depend on the shape of the transverse profile for a hard diffraction process of interest and on the kinematic range available for soft parton evolution, which is influenced, in turn, by the kinematics of the hard process. For example, we observed $\simeq 40$% difference for the noneikonal suppression factors between the cases of single and central diffraction. Consequently, our results can not be directly applied to other hard diffraction reactions.

The main suppression mechanism for hard diffraction, related to elastic rescatterings of constituent partons of the colliding protons, is defined by the interplay between the shape of the inelastic profile for general *pp* collisions and the transverse profile for a diffractive process of interest, as already demonstrated previously [1, 3, 11, 17]. On the other hand, it appears to depend sizably on color fluctuations in the proton, which thus introduces a significant model dependence for calculations of the RGS probability. Reversing the argument, experimental studies of rapidity gap survival in hard diffraction can provide an insight into the nonperturbative structure of the proton.

## Footnotes

1 Note, however, the arguments of Ref. [17] concerning a suppression of contributions of inelastic intermediate states.

2 It is noteworthy that the integrated parton density is, however, lower for Fock states of smaller size [11, 36].

3 To simplify the discussion, we neglect here Pomeron “loop” contributions, exemplified by the last graph in the rhs of Fig. 2, the complete treatment being described in Refs. [24, 31].

4 Strictly speaking, Eq. (8) contains also the contribution of double diffraction, corresponding to a dissociation of the projectile proton into a low mass hadronic system, in addition to the formation of a high mass state on the target side (see the discussion in Refs. [24, 39]). Similarly, Eq. (5) accounts also for situations when the projectile or/and target protons are excited into low mass hadronic states, in addition to the formation of the central diffractive system.

5 As mentioned above, in our discussion we neglect for simplicity the contributions of graphs containing Pomeron loops. In numerical calculations, presented in Sect. 3, we use the complete formalism of the QGSJET-II model, Pomeron loop contributions included.

6 Let us remind that in the current approach only rescatterings of soft ($|q^{2}|<Q_{0}^{2}$) partons are taken into consideration.
